# INSTRUCTIONAL MANUALS OF BOUNDARY‐WORK: PSYCHOLOGY TEXTBOOKS, STUDENT SUBJECTIVITIES, AND DISCIPLINARY HISTORIOGRAPHIES

**DOI:** 10.1002/jhbs.21791

**Published:** 2016-05-06

**Authors:** IVAN FLIS

## Abstract

This article aims to provide an overview of the historiography of psychology textbooks. In the overview, I identify and describe in detail two strands of writing histories of introductory textbooks of psychology and juxtapose them to provide an integrated historiography of textbooks in psychology. One strand is developed by teachers of psychology—first as a general approach for investigating textbooks in a pedagogical setting, and then later upgraded into a full history of psychology textbooks in America. The other strand follows a more familiar perspective of historians of science and historians of psychology who build on various post‐Kuhnian and post‐Foucauldian perspectives on textbooks. I make an argument for integrating these two views for a more comprehensive historiography of textbooks in psychology, recasting textbooks as objects of research and sources that are interesting sui generis for historians of psychology in their investigations.

## Introduction

In the past years, there has been a boom in the history of science pedagogy, and with that, textbook history. Kathryn Olesko (2006) unequivocally states that “[t]he historical study of science pedagogy has of late experienced a renaissance, and with that, a revolution in perspective” (p. 863). The articles in the volume of *Science & Pedagogy* for which Olesko wrote the above‐cited commentary attest to this change in perspective and focus, bringing together a collection of studies on the history of scientific and technological textbooks on the European periphery. As Bernadette Bensaude‐Vincent (2006) argues in the same volume, science teaching in general is interesting for historians “[n]ot only because it is indispensable for training new generations of scientists or because it enriches our view of science as a social and cultural activity, but also because it determines the disciplinary partitions of scientific knowledge.” She continues with saying that textbooks in particular “are sorts of archeological traces of former regimes of knowledge” (p. 668). Textbooks are not just repositories of uncontroversial facts used to disseminate them outside of tight‐knit scholarly communities—they can also serve as epistemological and institutional catalysts.

Olesko, and to a certain extent Bensaude‐Vincent, argue that the once‐exciting interpretative frameworks of Thomas Kuhn, Jerome Ravetz, and Michel Foucault have been eclipsed, at least in the case of the history of science pedagogy. Olesko (2006) states: “[U]nderstanding pedagogical experience is something greater than the sum of institutional and intellectual history” (p. 871). The gloomy perspective formed at the intersection of Foucault's disciplining of disciplines in *Discipline and Punish* and *The Order of Things* on one hand, and Kuhn's textbook science and its role in paradigmatic establishment of “normal science” on the other, is too sweeping and too insensitive to the particulars of what was actually happening in science classrooms in the eighteenth, nineteenth, and twentieth centuries. We need not go far to look for an example of the rich context these perspectives tend to stamp out, for example, Andrew Warwick's (2003) excellent study of the role of the pedagogical context and its tools in the rise of mathematical physics at Cambridge.

Science pedagogy, in particular concerning textbooks, is also relevant for history of psychology as a discipline. In this article I will focus on the historiography of introductory textbooks in psychology, highlighting the differences in the approaches taken by psychologists with those taken by historian‐psychologists. I aim to show that these textbook historiographies are particularly interesting as a space where genres of different communities meet to describe the same things—the textbooks used in introductory university courses in psychology—but the resulting descriptions are quite different. We can see the different agendas psychologists and historian‐psychologists project into their narratives, and in turn, the object constituted by those narratives is quite different. The difference results in a divergence in historiographies—two parallel streams of thought talking about introductory textbooks in psychology.

Psychology textbooks are not only interesting because of the divergence in historiographies talking about them, but also because of the particularities of textbooks in psychology versus other natural or human sciences. In psychology, textbooks are not only actual physical artifacts containing the partitions and classifications of previous (potentially eclipsed) knowledge systems, they are also a place for the expert to construct new subjectivities for the student (Morawski, 1992, 1996). This is quite particular to textbooks in the social sciences, and especially so in psychology. The authorial voice of the psychologist attempts to offer a different subjectivity to the student than the one experienced in the everyday: A scientifically constructed one.^1^ In effect, the twentieth century introductory textbook in psychology is an attempt made by psychologists to offer what are currently legitimate concepts of human psychology to the student. These new concepts aim to be the furniture of the students’ private (and idiosyncratic) psychological reality. The scientific account attempts to supplement, or even in a radical reading supplant, the commonsensical one—the one each of us has access to by the virtue of being human beings. This is especially the case in the textbooks found at the end of the nineteenth century, when psychology as a discipline is establishing itself. This makes psychology's textbooks particularly interesting as sources—of disciplinary partitioned regimes of knowledge that construct subjectivities. This construction of subjectivity also presupposes a particular epistemology used in fact making in psychology's textbooks that is not seen in other disciplinary introductory texts (Smyth, 2001a, 2001b, 2004).

The goals of this article are to describe two approaches to the histories of introductory textbooks in psychology—one produced by psychologists writing about textbooks—what I call the received view; and the alternative view by psychologists‐historians (Vaughn‐Blount et al., 2009) and historians. The received view is fashioned from a large number of articles published in the journal *Teaching of Psychology*, and then later synthesized into a historical account in Weiten and Wight's (1992) text; while the alternative view is discussed mostly through the work of Jill Morawski and Mary Smyth. I am primarily interested in the historiography of introductory texts used in undergraduate courses teaching general psychology, but the investigation of these books is unavoidably tied to other genres of textbooks—subdiscipline‐specific ones (e.g., social, developmental, abnormal psychology textbooks), or textbooks in methods and statistics.

In comparison to a renaissance of history of science pedagogy and history of textbooks as a broader trend, however, historians of psychology have still mostly avoided textbooks. There is some historical research using textbooks in psychology—some episodes in the discipline's history are entangled with using textbooks as sources—the case of Ben Harris's investigations (2011, 1979) of Watson's *Little Albert* experiment come to mind. The same goes for Stam, Lubek, and Radtke's (1998) investigation of the particular view on the Milgram's experiments produced and ossified in social psychology textbooks. An even more popular genre of textbook research among psychologists is investigating their biases (Brown & Brown, 1982; Winegard, Winegard, & Deaner, 2014) or even just examining them for general inaccuracy and errors (Steuer & Ham, 2008). This, however, is more contributing to the image of the denigrated faulty textbooks and their flawed accounts of science (Morawski, 1992) than to textbook historiography. For a more historical perspective, Thomas Teo's (2007) study of German psychology textbooks in the beginning of the nineteenth century is a breath of fresh air. Andrew Winston's studies on textbook definitions and redefinitions of psychological experiments should be mentioned here (1988; Winston, 1990; Winston & Blais, 1996; MacMartin & Winston, 2000; Winston, 2004), as well as his study on the changes in presentation of race and heredity in introductory textbooks (Winston, Butzer, & Ferris, 2004).

The author that does stand apart in this canvassing of historical work on psychology's textbooks is the previously mentioned Jill Morawski (1992) with her almost programmatic article in the *American Psychologist* on how textbooks of psychology create subjectivities, and her later full analysis of the rhetoric in textbooks in the beginning of the twentieth century (Morawski, 1996). Coupled with a series of Latourian readings of psychology's textbooks from the second part of the twentieth century by Mary Smyth (2001a, 2001b, 2004), and the already mentioned investigations of the experiment through psychological textbooks by Winston (1988; Winston & Blais, 1996; especially the discourse analysis in MacMartin & Winston, 2000), Morawski's approach offers a solid basis for a historiography of psychology's textbooks that fits nicely in the current discourse espoused by Olesko and Bensaude‐Vincent. Maybe it is not a boom evidenced in other disciplinary histories, but it is a definite presence that must be mentioned.

However, the received historical view of psychology's textbooks is not to be identified with the work of Morawski, Smyth, Winston, Stam, or Teo. As the body of this article will show, historiography of introductory textbooks in psychology is quite peculiar. I call it the psychologists’ received view of textbooks and attempt to describe it in detail. For the most part, the received view does not delve in the disciplinary partitions found in textbooks. Disciplinary formation and organization of knowledge are not the research objects of the received view. Instead, the type of historical research gathered around the received view on textbooks is precisely an extension of these intradisciplinary negotiations. In simpler terms, the psychologists’ methodologies and ways of thinking have expanded from humans and rats to include textbooks. Psychologists have fashioned a received (standard) view of their textbooks that does not have much to do with the work of historians of science. In this article, I aim to describe this received view through the example of Weiten and Wight's (1992) large chapter on the history of American textbooks, and a large sample of articles on textbooks published in the journal *Teaching of Psychology*, on which Weiten and Wight build their chapter. Then, this standard view on textbooks will be juxtaposed to that of Morawski and Smyth, and as a conclusion, an integrated approach to the historiography of textbooks in psychology will be suggested.

This integrated view will not only suggest the physical textbooks as a historian's object of research in the case of psychology, but the extended literature about textbooks written by psychologists. Disciplinary negotiations and boundary‐work which is interesting for a historian of psychology does not only happen on the pages of introductory texts aimed at freshmen, but also in the wider literature of commentary and research on textbooks done by psychologists. Put like that, the whole genre of writing about introductory textbooks provides an entry point for exploring psychologists’ unwritten methodological horizons in general. The way they approach textbooks is also indicative of the way psychologists approach other subjects and research questions, at least in the second part of the twentieth century.

## The Received View of Psychological Textbooks: From Whose Vantage Point?

The received view of textbook history in psychology is produced by a different community of scholars who have nothing to do with tracing disciplinary formation in a way historians like Bensaude‐Vincent or Olesko aim at, or identifying the construction of subjectivities like Morawski does. This is not to say that there is an essential tension between this psychologists’ received view and the historians’ discourse on textbooks sketched thus far—it is more like a chasm of silence. The received view was produced as an unexpected amalgamation, a sort of a big overview that tried to create a historical discourse out of the collective research of a number of scholars publishing on textbooks in the journal *Teaching of Psychology*. The view took actual shape as Wayne Weiten and Randall D. Wight's (1992) chapter on textbooks in the APA volume *Teaching of Psychology in America: A history*.

Other than providing the most detailed chronology and bibliography of English language psychology textbooks ever published, it also provides a particular way of understanding what a textbook as an object of research is, and what it can offer us. The textbook and its history, as described by Weiten and Wight, stand in stark contrast to the discourse sketched from the work of Olesko, Bensaude‐Vincent, Morawski, and Smyth. Incidentally, the one time (out of the two) when Weiten and Wight's chapter cites Jill Morawski's work is to corroborate the authors’ claim that fellow academics look at textbooks with “suspicion and scorn” (Weiten & Wight, 1992, p. 487). Her substantive contribution to textbook historiography is ignored.

Calling the appearance of the received view of psychological textbooks an unexpected amalgamation without an explanation might be misleading. The production of this view was not unexpected in how it was written or researched—its meticulous and large bibliography attest to that. The unexpectedness was in its entrance to the scene of history of psychology, where it appeared in a chapter in an edited volume on the history of teaching of psychology, tying together a literature that was probably never imagined to produce historically relevant knowledge. The chapter was the crest of hundreds of articles on textbooks published in *Teaching of Psychology*, which in the way they are written, in the points they argue, and in the audience they address do not have much to do with the typical questions historians ask or the answers they hope to get.

To avoid ambiguity—and scholasticism in dispelling it—this point has to be carefully presented because it relates to the crux of the argument in this article. It boils down to the juxtaposition of two histories—one produced for historians and the other produced for psychologists‐turned‐historians.^2^ This is not to say that one of those is “good” history and the other “bad,” just that they serve different goals and different audiences. As Bert Theunissen (2001) argues from the perspective of a historian of science—there is no reason why scientists’ histories of their disciplines should be “bad” histories by default “[b]ut in practice, scientists’ histories do tend to differ quite substantially from the kind of history written by professional historians of science” (p. 148).

Theunissen draws the distinction between scientists and historians, and the aims they have in writing histories. In the case of textbooks, this distinction is complicated by the fact that the group juxtaposed to the historians is not just scientists, but science *teachers*. Teachers of psychology have a particular goal when they use history—the tension between the history for history's sake on one end and history used to teach on the other leads to Stephen Brush's (1974) complex point in the article *Should the History of Science Be Rated X?* that problematizes the practice of exposing students to narratives that “threaten” scientific objectivity. I argue that this tension is not only manifested in the history found *in* the textbooks, but also in the history *of* those textbooks. The ground for criticism is not only the familiar Kuhnian point about textbook histories—the grand simplifications found in textbooks instead of histories—but also the contextualization of the sequence of these grand simplifications. In other words, textbook histories have a history.

Teachers of psychology are the most common users, but also often producers, of the said textbooks. It follows then that they have a goal of carving a niche for the history of textbooks. A niche that should be defended against the scientists’ scorn toward their inaccuracies and foibles but also against the historians’ approach that has the potential of breaking down the discourse in the textbook in order to look for traces of disciplinary formation, contingencies outside of the discipline, or negotiations of objectivity. Negotiations of objectivity, as Brush warns us, are especially perilous when they find their way into the classroom.

Weiten and Wight's goal—in what I call here the received view of textbooks—is to describe how we ended up with modern textbooks, or in their words, the development of these “portraits of a discipline.” In comparison, potentially both the historians’ and the scientists’ view on textbooks have a deflating note, looking down on them from the pedestals of historical contingency (historians of science) or uncompromising objectivity (practicing scientists). The carving of this niche in the face of scientists’ derision is evident from the way Weiten and Wight (1992) conclude the chapter: “[I]t seems shortsighted to evaluate introductory textbooks by the canons of scholarship applied to journal articles” (p. 488).

Opposing it to the historian's perspective is more complicated than just reading it out of their conclusions. After all, Weiten and Wight's chapter styles itself as a historical account. To this end, their historiographical approach will be described in detail, with an analysis of the constituency it represents. Calling it a constituency is a conscious choice, trying to avoid terms that might lead us to over‐interpret, terms such as *Denkkollektiv* (Fleck, 1979), invisible college or even a research community (Crane, 1972), or Kuhn's own members of a community gathered around a paradigm (1962/1970). Instead, minimal interpretation will be allowed as to what kind of a community is formed by the authors gathered around Weiten and Wight's chapter on textbooks. The emphasis is on their perspective on textbooks, the received view, as it is fashioned from the interaction of the producers’ know‐how and the audience's expectations.

## Weiten and Wight's Portrait of a Discipline Gleamed from Textbooks

The 1992 volume on the history of teaching of psychology in America was imagined as a celebratory, but crucial, contribution to the growing scholarship on the history of psychology. As Charles Spielberger (1992), the APA president at the time, aptly put it in the foreword of the volume: “Although history of psychology is well documented in numerous books and articles, relatively little attention has been directed to examining the history of *teaching* of psychology” (p. xvii). The volume was aimed to fill this lacuna in the historiography of psychology, and provide support and incentive for future research. Indeed, the editors themselves call it a baseline: “Although not meant to be the final word, this collection is reasonably exhaustive and thought provoking, raising questions that provide an impetus for further analyses in the field and a baseline from which to trace developments into the 21^st^ century” (Puente, Matthews, & Brewer, 1992, p. 7). They go on to conclude that this will not only add to history, but also to better teaching of psychology in America. Weiten and Wight's chapter, then, is aimed to fulfill this goal in covering the role of textbooks in the history of teaching of psychology. The tone of the volume and its institutional endorsement is what immediately rings of a received view—it announces that the chapters contained within spell out what is what in the history of teaching of psychology.

As Weiten and Wight put it themselves (1992): “The scholarly literature on introductory texts remains sparse, and the few articles available typically focus on one text or author. We hope our chapter will help to fill this void in psychology's intellectual history” (p. 454). Their chapter consists of two large sections—the first one providing a sophisticated and detailed chronology of more than a century of psychology's textbooks, with a basic periodization and extensive bibliography, and the second giving a quantitative analysis of the textbooks in this chronology. The chronological overview is quite a valuable contribution to the historiography of textbooks in psychology and I will shortly examine it in more detail first.

### The Definite Textbook Chronology

The chronology allows for a periodization of textbooks in line with the development of American psychology as a discipline—from the textbooks used in courses of moral philosophy in the last decades of the nineteenth century, to the transition to the “new psychology,” the period of conflicting schools and theoretical eclecticism, and finally to the rise of the student‐oriented texts in the 1930s–1940s and the encyclopedic texts that were the prerequisite for the homogenization and standardization of the modern textbooks since the 1970s. As an overview, this periodization serves its purpose in providing a factual timeline of publications to be analyzed. However, despite its dearth, the periodization is not without its problems.

The first question that a periodization and a classification should engage with is demarcation—what makes a psychology textbook? Is it an introduction to a self‐standing academic discipline, or to a range of topics investigated by particular scholars? In Weiten and Wight's chapter these questions are not addressed. Was a book used in the classroom taught by American mental philosophers or the teachers of Scottish “commonsense” philosophy (Fuchs, 2000) an introductory textbook to psychology, or did it take the new psychology imported from Germany and the laboratories proliferating with that migration to constitute the first textbooks? When did the shift from an introduction of psychological topics to an introduction to a (empirical!) discipline happen? These are precisely the kind of questions that should anchor the overarching chronology of psychology's textbooks, but they are just skirted over by Weiten and Wight (1992) with the sentence: “Textbooks intended to introduce college students to the field of psychology emerged gradually out of the work in moral philosophy during the 19^th^ century” (p. 455). What can be read out of this is an essentialist conception of psychology as a discipline.^3^

The particular kind of history of textbooks created by Weiten and Wight comes in full force in the second section of their chapter, which is the analysis of the meticulous chronology they constructed. To avoid being pedantic, and also to alleviate the potential criticism that my interpretation of the standard view was built on a single text written by two psychologists; the following section of this article will try to explore Weiten and Wight's received view through the connected network of articles produced by their audience. There is a small community of scholars expanding out of the *Portrait of a Discipline* into a citation network. This citation network creates an academic ecology in which the received view flourishes, and for which it was crafted in the first place.

## How to Identify an Audience of a Chapter in a Book?

The audience was identified in a number of ways—it was actually triangulated through three separate approaches: looking at the reference list of the chapter, conducting a search in the Web of Science database, and identifying the publisher of the journal. All three things pointed, more or less, to a single journal: *Teaching of Psychology*.

The APA Division 2—the division on teaching of psychology that sponsored and organized the writing of the volume containing Weiten and Wight's chapter publishes a journal. The history of the said journal is also a subject of interest for the volume editors, so a full chapter is dedicated to its founding and historical development (Daniel, 1992). The division shares the name with their journal—*Teaching of Psychology*. Not surprisingly, looking at the reference list of Weiten and Wight's chapter, that journal plays a prominent role there too—at a cursory count, publications in *Teaching of Psychology* are cited about 30 times. This is of course no definite proof that the authors of those publications are the intended audience of the chapter, but it does imply a certain community of scholars writing about the same topic.

Going to Web of Science and searching for the topic “psychology textbooks” one journal came under spotlight—by now, no surprise there, it was *Teaching of Psychology*. The journal ranked first considering the number of relevant publications, containing 145 articles on psychology textbooks. The next in line was *Psychological Reports* with just 27.

By this point, the idea that a constituency of the standard view could be traced through the publications on textbooks in *Teaching of Psychology* took form. Instead of just using the 145 articles pertaining to textbooks in *Teaching of Psychology* that were found in the first search of Web of Science, the search was redid on that particular journal. My reasoning in this was that maybe some of the publications were missed, and will have been located with a finer‐grained search of that particular journal. In total this identified 3,174 entries as the whole corpus of the journal. When the full corpus was refined to only those including the word “textbook” in their keywords, titles, and abstracts, we arrive at 184 publications. Then, the reference lists of those 184 articles were analyzed using *CitNetExplorer*, a program developed by Nees‐Jan van Eck and Ludo Waltman (2014a) specifically for citation network analysis.

### A Look at the Received View through Citations

*CitNetExplorer* directly builds on Garfield's notion of *algorithmic historiography* which is an approach that tries to implement bibliometric tools of citation analysis in historiographical studies; and Garfield developed a program for such analyses. As Garfield, Pudovkin, and Istomin put it in the description of the said software: “[*HistCite^TM^*] facilitates the understanding of paradigms by enabling the scholar to identify the significant works on a given topic … it provides a graphic, genealogic presentation of citational links between them” (2003, p. 400). *CitNetExplorer* is a more sophisticated program offering similar functionality—it is basically used for citation analysis of literature, for example, “for studying the development of a research field over time, delineating research areas, studying the publication oeuvre of a researcher, [and] literature reviewing” (Van Eck & Waltman, 2014b). In our case, it was used to analyze the literature about psychological textbooks in *Teaching of Psychology*.

**Figure 1:**
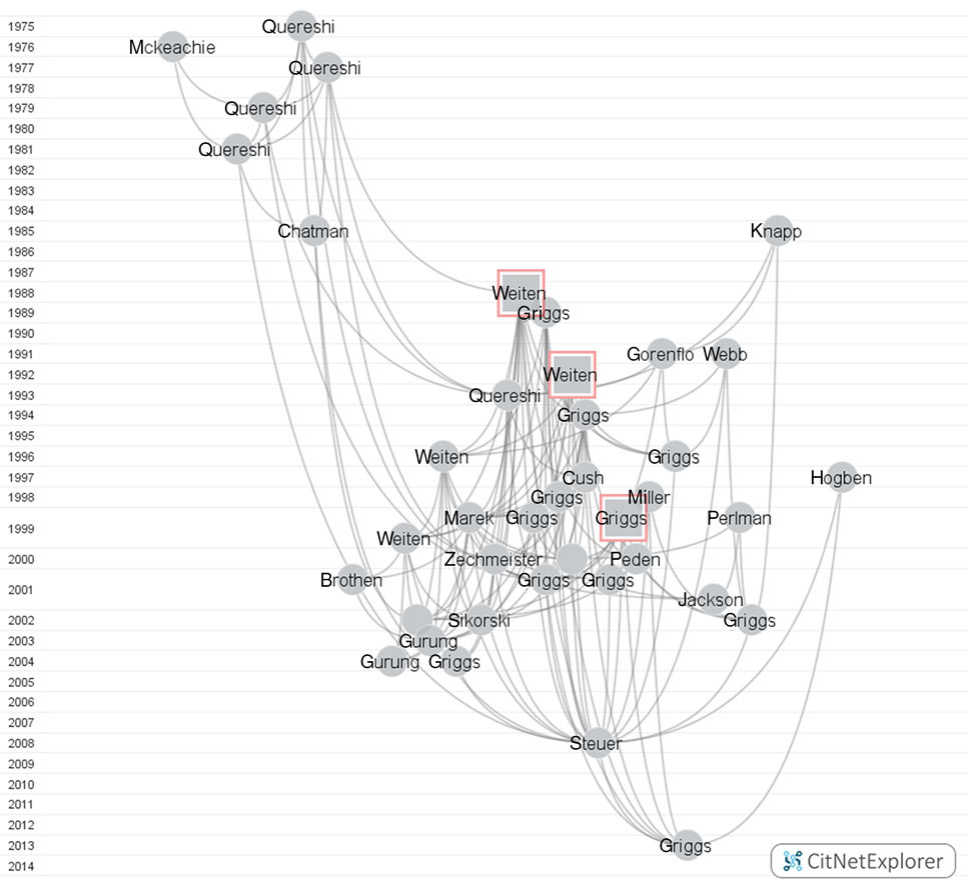
Teaching of psychology citation network.

*CitNetExplorer* extracted references from the reference lists of the 184 articles in *Teaching of Psychology* that were identified to pertain to textbooks, and mapped the references to the articles in *Teaching of Psychology*, and to other publications if they were cited 10 or more times by the said 184 articles. The product of this mapping can be seen in Figure 1, for clarity only including the 40 articles with the highest citation scores. This represents the framework in which authors publishing in *Teaching of Psychology* write and think when they discuss textbooks. Keep in mind; it is the network of the references of those 184 articles, not of the articles themselves!

The vertical axis represents the publication years. The closer the publications are horizontally, the bigger is their citation relation. Every circle is a single publication, marked with the name of the first author, while the curved lines represent citation relations between the publications. Citations point in the upward direction, meaning that the cited publication is always located above the citing publication. We can identify the central publications in the network according to internal citation scores—the number of times a publication was cited within the network. The three most cited publications are marked as squares instead of circles, and those are: Weiten and Wight's *Portraits of a Discipline* (1992; the lower square *Weiten* in the figure), Weiten's *Objective Features of Introductory Psychology Textbooks as Related to Professors’ Impressions* (1988; the upper square *Weiten* in the figure), and Griggs et al.’s *Introductory Psychology Textbooks: An Objective Analysis and Update* (1999; the only square *Griggs* in the figure). The full list of publications in the citation network can be found in the Appendix, with the first 40 publications from the appended list represented in Figure 1.

All the articles in the Appendix were inspected to ascertain how many of them are actually about introductory psychology textbooks, versus psychology textbooks in general. This was done by examining the titles, and in unclear cases, abstracts and the articles themselves. Out of the 188 articles in the citation network, 115 deal exclusively or in part with introductory psychology textbooks and courses—others deal with subdiscipline‐specific textbooks or other topics relevant to teaching of psychology. It is safe to conclude that the bulk of the focus on textbooks in psychology, as far as *Teaching of Psychology* goes, is oriented toward introductory textbooks.

The interesting thing about this citation network is that it represents the articles and publications on textbooks cited by the authors publishing in *Teaching of Psychology*. So it is not only supposed to represent the articles they publish in that particular journal, but *all* the articles published across a number of journals that might cover psychology textbook research (if they were cited often enough). Well, in principle that is true, but it actually does not cover anything else but *Teaching of Psychology*. The network is self‐contained.

The closed loop is obvious when one looks at where do the publications in the network come from: all except of one are published in the journal *Teaching of Psychology*. The one that was not published in that journal is precisely the Weiten and Wight's chapter analyzed before, sitting there in the middle of the citational mentalscape of the authors publishing about psychological textbooks in *Teaching of Psychology*.

## Description of a Constituency—Quereshi in the 1970s and the 1980s

Weiten and Wight sampled textbooks from 1890 to 1990 for their chapter, and compared them on a set of quantified variables—text variables, measures quantifying topical coverage, topical organization, book size, illustrations, citations, and references. This is treating the textbook as an object of very familiar quantitative methodologies employed by psychologists in other research areas of psychology. The psychologists’ toolbox was applied to an object of research that was made of ink and paper, instead of the objects being—like in that old and weary joke—freshmen and rats.

In our displayed citation network, the oldest consistent example of an approach similar to Weiten and Wight's can be found in the articles published by M. Y. Quereshi. The content analysis approach pursued by him opened up the field of introductory textbooks to psychologists applying their usual methods of research. Quereshi and his various associates published four articles from 1975 to 1981 on content analysis and its various applications to textbooks (Quereshi & Zulli, 1975; Quereshi & Sackett 1977; Quereshi & Buchkoski, 1978; Quereshi, 1981) that were caught into our citation network as highly cited.

In their first content analysis (Quereshi & Zulli, 1975), the authors stress how important it is to learn about the content of introductory texts, considering they are the biggest influence on teaching of psychology alongside the instructor. Stressing the important role of textbooks seems to be a crucial element of the received view. For the psychologists endorsing it, research on textbooks offers insight into the role and function of classroom education both for the future profession and science. One of the common arguments to support this assertion by the community gathered around the received view are the enormous sales figures, which by the force of their sheer volume assert their importance. For example, Steuer and Ham (2008, p. 160): “Informed estimates suggest that annual domestic expenditures on all psychology textbooks reach $160–200 million (S. Scarrazzo, personal communication, July 11, 2005). Such figures imply that introductory textbooks in combination with those used in upper level undergraduate courses constitute a substantial—and potentially massive—part of the education experience of most psychology students.” The other support for the importance of textbooks Steuer and Ham mobilize is Kihlstrom's (2010) survey of “fondly remembered textbooks” on three professional psychology listservs.

Are these compelling arguments for the suggestion that introductory textbooks have a relevant impact on future psychologists, or is this kind of impact reserved for the more advanced handbooks? It is difficult to tell. Winston (1990) questions it when comparing the impact of Woodworth's famous Columbia Bible on experimental psychology to his more popular (by sales) introductory texts for lower level intro courses. As Winston (p. 394) puts it: “Despite these enormous sales [of Woodworth's introductory level *Psychology*], it is difficult to gauge the influence of an introductory psychology course and an introductory text on future psychologists. In contrast, it is in the experimental psychology course that students learn how to *do* research.” Considering the above, the shakily supported (almost taken‐for‐granted) belief that basic introductory texts do exert influence seems to be an important component of the received view.

Coming back to Quereshi and his associates, we see that he tries to expand on previous studies on comparative readability and human interest by pursuing “an objective and systematic analysis of their [the textbooks] salient terms” (Quereshi & Zulli, 1975, p. 60). The authors’ interest is of the practical kind—they aim to provide insight about the content of textbooks that will be used by teachers in introductory courses at American universities and colleges. It is a given, then, that systematic content analysis produced by coding subject indices of 25 textbooks and conducting factor analysis on them, what Quereshi in effect did, will provide useful knowledge about textbooks. The function of the textbook is pedagogical, and Quereshi and Zulli are helping with the role textbooks take in teaching by providing more knowledge about them. That is the logic behind their research. What is the actual execution?

Using factor analysis, they cluster the 25 textbooks around 10 factors, based on the 2,648 terms that appeared in the subject index of more than one textbook. The main part of the article is an extensive explanation of the naming of the 10 factors they have obtained, identifying the similarities between the textbooks gathered around a particular factor. The result of this naming procedure is a taxonomy that topically groups the textbooks.

Even though Quereshi's factor analysis gives us a system of statistically derived categories for sorting textbooks, the procedure of naming the latent variable still involves a close‐reading examination of indices, tables of contents, and main bodies of the books to explain the organizing principle derived by factor analysis. When one takes into account the sophistication of this analysis in 1975, involving punch cards and IBM computers—the authors went through a lot of effort to gain an organizing principle that would have arisen by mere canvassing of the books—did we gain anything substantive from this conclusion? Some textbooks cover experimental psychology, others quantitative methods, and yet others focus on genetics. If we reason like this with factor analysis, would we not reason the same by just skimming the textbooks? We probably would have. In that regard, the article fulfills the function of structuring the way a teacher would choose an appropriate textbook for her class. Most importantly, it structures the choice *quantitatively*.

Introductory texts are just aggregators of information—some are focused on one kind of information, some on the other; some are more readable than others, but they all fulfill the same function. The implicit conclusion is also about the method, not about the textbooks—factor analysis is a suitable method to explore this pedagogical function of textbooks, by looking at their content.

The 1981 paper *Analytic Procedures for Selecting a General Psychology Textbook* represents the culmination of Quereshi's approach integrating findings and conceptions of the introductory textbook from his older articles in the cluster. It is also one of the blueprints for the received view of textbook research. The article presents analytical selection strategies the help instructors choose textbooks. The selection strategies are based on quantified variables the author connects with the textbooks in his previous publications—various measures of readability, human interest, the number of pages of text, pages of index, etc. As Quereshi (1981) himself describes the purposes he wishes to achieve: “The present study was done to carry out further analyses, as described below, in order to attain the major objective of devising analytic procedures for textbook selection” (p. 143). It is not only about devising a selection procedure, but about devising an analytic one, which for all intents and purposes means quantified.

The above‐described cluster of articles by Quereshi and associates provides a glimpse into the beginnings of consistent application of psychological quantitative methodology to exploring introductory textbooks. The list of articles on textbooks is far from exhaustive (e.g., Gillen, 1973; Harari & Jacobson, 1984), and far from fully contained by the citation network presented in Figure 1. I do not claim Quereshi and his publications provide a direct model for all the other applications and papers published on the topic. They do provide a case study, and by exploring them in detail we immerse ourselves in the view psychologists consistently endorse and develop when thinking and writing about textbooks. By critically reading the articles and trying to ascertain the goal the authors tried to achieve we are taking a glance at how psychologists went about solving practical problems, like those of textbook selection. What later arose out of the solutions to these practical problems, specifically in Weiten and Wight's chapter, was a history of psychological textbooks.

The same story as the one derived from Quereshi's content analyses could be told out of most articles in the citation network. For example, Weiten's (1988, p. 10) article trying to “…gather normative and comparative data on introductory texts and to explore how professors’ impressions of these texts may be shaped,” or Griggs's report on the change in percentages of certain topical coverages from the 1980s to the 2000s (2013). It all points to a textbook as a quantifiably dissected research object; a research object that is thriving in *Teaching of Psychology*.

## The Alternative to the Received View

So far I framed the received view of textbooks as objects of research defined by the “usual psychologists’ methodology” and conceptual horizon; textbooks as classroom‐specific aggregators of information mostly without a wider reflexive content relating to a particular discourse, disciplinary culture, and disciplinary identity. They are pedagogical, they are quantified, and they need to be optimized. This view of textbooks is standardized by a constituency of scholars that exists at least since the 1970s, the view has a journal outlet for publications, and is the baseline for a codified and described history in an institutionally endorsed tome. The historical part was most likely unintended—I do not think Quereshi or most of the other authors in the citation network thought of their research as writing a history of textbooks. They were looking for objective characteristics of textbooks. As psychologists are often wont they looked for objective characteristics of their object of research but what they found was a history (Gergen, 1973). What Weiten and Wight did was explicitly recasting this methodological approach as history.

Calling something usual, like I did with psychologists’ methodology, calls for more explanation. The usual psychologists’ methodology mentioned here is what Kurt Danziger (1996) calls arithmetization—psychologists turning their objects of scientific interest into quantities represented by variables, “information in a form that lends itself to counting” (p. 30); and by doing so their methodological toolkit becomes an appropriate way to talk about textbooks. The discourse of these techniques then defines the object of research—the discourse of the research practice, its variables and percentages and factorially derived categorical structures; *becomes* what a textbook is. The standard view performatively constitutes the historiographical objects of research, in other words, Danziger's arithmetization totalizes the textbook as an object with particular characteristics.^4^ The arithmetization happened already with Quereshi, making textbooks suitable research interests for psychologists, and then 20 years later it was refashioned into a history of textbooks by Weiten and Wight.

Considering the above, an alternative performativity—authors outside of the constituency sketched around the standard view—is sought to allow for an all (or better said, more) encompassing historiographical object, a textbook situated in a time, in a place, and in a community of users and producers; a textbook that tells interesting stories not only if we want to answer questions about the percentage of particular topical coverage through the decades (for example), but also if we want to ask reflective and substantive questions about psychology as a discipline and as a science. Basically, a textbook fit for a fundamentally different audience than the one imagined by the received view. In the following part of the article, the alternative will be described through the work of Morawski and Smyth, identifying its four most salient differences when compared to the received one.

### Looking at Textbooks as Instructional Manuals of Boundary‐Work

A detour from the standard view has to start with discursively locating the textbook not only for its quantitatively represented content and the optimization of that content for a classroom, but for it as an object operating in multiple contexts at any given time. Jill Morawski (1992) approaches textbooks with this seemingly nonpedagogical question when she is trying to “ascertain scientists’ representation of their work to nonscientists” (p. 161). Not only is a textbook a pedagogical tool in the commonsensical understanding as an assemblage of content that needs to be presented and then learned, it is also an object serving as grounds for exchange between two distinct groups—experts and laymen. It is an instructional manual of boundary‐work (Gieryn, 1999) for psychological knowledge. Textbooks are “textual artifacts,” as Morawski calls them, which tell us something about the actors’ own understanding of psychology, with quite an evaluative judgement included in that from the actors’ side. The evaluative judgment being as follows: Because certain content is included in a textbook, it must be a part of a certain canon. It acts as a border between the laymen and the expert, trying to transfer knowledge from one side to the other: from the expert to the laymen, that is, from the professor to the student. At the same time, calling it an instructional manual of boundary‐work, I mean it actually *does* boundary‐work on what psychology is through how it is describing the discipline. The negotiations of borders in discipline‐making are frozen in textual form.

It is not far‐fetched to say that practical examples of textbooks as conceptual workhorses in boundary‐work abound—here is an example from the 4th edition of Hilgard and Atkinson's *Introduction to Psychology* (1967, p. 7): “The emphasis in this book will reflect the general orientation of American psychology today, which seeks to place the study of behavior in the context of natural science, recognizing man's affiliation with other biological organisms, and hence tracing continuities between man and other animals. Such an orientation need not deny man's uniqueness where such uniqueness is demonstrable.” This quote is taken from the introduction of the book, where it is part of setting the stage—explaining the position and role of psychology in comparison to other disciplines. What this shows is that in psychological textbooks the boundary‐making becomes quite explicit, for example, in the introduction of Clifford Morgan's (1956) textbook:

“As science increases in pace and widens its grasp, it becomes harder to find the borders of demarcation among the behavioral sciences. Actually, there are no boundary lines, only no man's lands of unexplored territory or overlapping domains in which scientists of different labels work side by side. In the general area of behavioral science, psychology is a kind of meeting ground for the natural sciences such as physics, biology, and physiology, and the social sciences such as sociology, economics and political science.” (p. 5)

Reading textbooks as boundary‐objects has its merits, but it is far from exhausting their potential as objects of historical studies. Looking for constructed subjectivities these boundary‐objects serve to propagate, like Morawski does (1996), sets the bar even higher. One can ask here: Is psychology a science of subjectivities, or of objective laws of human behavior and mental processes? Asking this question is precisely what a thought‐provoking historical study of textbooks would motivate, and potentially answer.

### Identifying and Describing the Experts’ Construal of Subjectivity

Looking at psychological textbooks in particular, Morawski sees them as settings for introducing constructed subjectivities—various conceptualizations of the human mind and human experience that are generated by psychologists, but might seem quite artificial to the people reading them. The psychologists’ account of the mental and behavioral are not necessarily in lockstep with what we experience every day—this needs mediation, and textbooks serve that goal. Psychologists’ constructions of human experience need hard work to expand into the networks of psychological makeups of real people—the personalities, perceptions, intelligences, attitudes, and other concepts psychologists develop do not suddenly appear in people's minds as self‐referential terms to describe our own personal psychological experience, there is hard work involved in this conceptual expansion (see Latour, 1999; Latour, 2005). Hard work done in part by textbooks. As Morawski (1996) puts it:

“In advocating a world that takes subjectivity as an object with characteristics not unlike the ‘natural’ objects of other sciences, and simultaneously claiming superior knowledge of subjectivity, textbook writers had to address and engage the very subjects whose own subjective experiences were to be radically reinterpreted by the science. Textbook authors, then, faced the apparent paradox of denying certain subjectivities while attempting to enlist those very subjectivities in the project of scientific psychology.” (p. 146)

This construction of subjectivities is conducted in a particular way—a way of fact making, basically of persuasively generating knowledge. The rhetoric of the textbook encompasses various strategies of fact making that are quite particular for textbooks in psychology, and we will take a look at them through the work of Mary Smyth.

### Exploring the Role of Textbooks in the Construction of Facts

Mary Smyth's research on textbooks expands the post‐Kuhnian perspective; for her “[t]he textbook, in removing context, is not distorting the history of science, but is actually part of it, and deserves to be studied as part of the continuing process of construction and reconstruction in science (Hacking, 1999)” (2001a, pp. 609–610). She points to an interesting tension—part and parcel to scientific research is decontextualizing and simplifying the object under study. This does not make scientists and the research they conduct unsuitable for historical research; on the contrary, it makes it particularly attractive. Why would such a thing make textbooks unsuitable for historians then? She includes textbooks as one of the building blocks of psychology as a discipline through their role in fact making, and tries to investigate the thorny path psychological facts travel in their last stage—not at their birth in the laboratory and their trip to the journals like Latour and Woolgar (1986) did—but on their trip from the journal to a textbook.

Smyth is on the hunt for autonomous fact statements in psychology textbooks, with the hopes of answering questions such as: Do the facts produced by psychologists follow the same trajectories as those described by Latour and Woolgar in the case of biology? Are the textual ecologies of those facts comparable between biology and psychology? In effect, is Latour's conception of facts applicable to a different science than biology, in particular, psychology?

In her article *Fact Making in Psychology: The Voice of the Introductory Textbook*, Smyth analyzes psychology textbook chapters on two topics—memory and social interaction (2001a). The conclusion of her analysis is quite provocative: Textbooks in psychology do not function as receptacles of facts like they do in biology or other disciplines. The representation of facts in psychology is different, and this is a consequence of a different fact‐making process. Psychological knowledge is presented with the evidence of its making. Unlike biology, where what signals knowledge is the obfuscation of the history of its making, psychologists employ the opposite strategy. Valid knowledge is designated by qualifications. As Smyth puts it: “Psychological evidence carries its knowledge with it,” (p. 628) and this can be extended even further to say that psychological knowledge qualifies as knowledge precisely because of the history of its making.

This is not done to legitimize the substantive fact (e.g., a particular model of memory) but to legitimize the way it was constructed—the methodology that made it possible for that substantive fact to come into being. The autonomous knowledge presented is not about substantive psychological phenomena, but about the ways of reaching and inferring these phenomena. Circling back a bit, this is similar to Quereshi and his textbooks—he is not saying that much about textbooks themselves, but more about the ways one can conceptualize them in a quantitative fashion.

Extending this argument to the extreme, psychological knowledge is then the method employed by psychologist whereas the phenomena, theories, models, and psychological constructs are just epiphenomena to mask the true epistemological claim. The qualifier (the modality in Latour's words) takes center stage, not in making an ontological claim about human psychology, but to make a strong epistemological claim that psychologists have methods to uncover relevant knowledge about the psyche at their disposal. The textbook is making a claim about the road to knowledge; the actual knowledge at the end of it is of lesser importance.

### Problematizing the Function of Textbooks as Vindicators of Psychology as a Science

In looking at how psychologists as textbooks authors go about presenting their science, Smyth tries to learn something about psychology as a discipline. She puts it in the context of Gigerenzer's surrogates for theories (1998) and Bazerman's (1988) behaviorist shift in how psychologists write and conduct their science. The practice of textbooks presenting results and studies with full references precisely fulfills the goal of the textbook author; the goal being the provision of epistemological legitimacy to the discipline where otherwise it would be questioned. The question if this legitimacy‐building strategy succeeds is something to be found outside of the textbooks.

To put it differently, the surrogates for theories as Gigerenzer calls them, in our analysis based on textbooks, is the discourse of an empirical psychological science. It teaches epistemologically, it disciplines and orders: “Always present evidence—this is the message about practice that the new psychologist absorbs from these textbooks. The paradigm is one of doing, not one of knowing” (Smyth, 2001a, p. 629). It also rings true of Danziger's suggestion that psychologists’ disciplinary discourse is actually the discourse of their investigative practice (1996). The structure of argumentation, the rhetoric the textbook author employs, is a model for a psychologist‐to‐be to learn how to argue her points in the future, be it as a researcher or as a professional. It teaches an interpretative and justified knowledge‐making culture for producing psychological phenomena.

Here, the pedagogical expands into disciplinary formation. In the pedagogical practice of textbook writing, literally in how they were written, we can see some of the ramifications on what students do later as psychologists. Thus, the textbook is not only an implement to teach on a theoretical level, but a practical model‐example.

Smyth ventures further in her analysis, trying to understand why the difference in the practice of writing textbooks between biology and psychology arose in the first place: “Psychology […] presents evidence in its textbooks to override the engagement of the reader's everyday knowledge. There is no explicit, direct indication that folk psychology or common knowledge is to be replaced […] yet the continual reference to evidence indicates that there is an argument with the reader going on, or the possibility of one. The reader is to be convinced that these accounts are reasonable, not told that they are so” (Smyth, 2001a, p. 632). The readers of a psychology textbook have direct access to their own psychological reality. They are directly at the source, and to be convinced that there is more to know about it than direct experience, something different than an autonomous fact statement is needed. An epistemologically superior method is required, and the hedging and the qualifications in textbooks show off precisely that. Echoing the Quine counterfactual read from Morawski's view on textbooks, the method in psychology is competing with common sense; it is not an extension of it.

This turns Latour's understanding of modalities, of hedging and qualifying, upside down. Modalities in psychology are employed to provide solidity for the claims, and in doing so, those very modalities are legitimized and unquestioned. The psychological claims in textbooks are a flood of evidence—they blunt opposition by empirical corroboration, a mob of studies and articles pregnant with experiments and sound research designs, signed with a name and a surname, and overtly supported by institutional and disciplinary affiliations. They are the heritage and the system empirical psychology leaves—the practice, not the knowing. And historians can explore the historicity of that practice through textbooks.

### The Virtues of the Alternative View

The alternative view clearly provides a different way of historicizing textbooks in psychology. We can link it to how Kurt Danziger (1990, 1997) approached investigative practice—he extensively used journal articles to situate his history of psychology's investigative practice in order to learn more about the discipline. Another route to historicizing psychology can be traveled by looking at textbooks, providing a view of psychology from a counterpart to investigative practices: the practices of teaching psychology. As in the example above, maybe Danziger's investigative practices and the pedagogical practices turn out to be one and the same, the building blocks of psychological discourse in (at least) the second part of the twentieth century. There is a timeline to these pedagogical practices, their development and change, which can be tracked through textbooks.

Smyth's investigation of fact making and Morawski's analysis of discourse in constructing subjectivities provide a framework for a diachronic perspective on textbooks. When did the practice of hedging and qualifying statements Smyth describes appear in textbooks? What was the referencing practice in the period Morawski investigates, at the turn of the century? In turn, what is the authorial voice in the later textbooks Smyth focuses on? These are all questions the historiography of psychological textbooks should engage with, but that have no place in the received view of most psychologists when they write about textbooks. With this short excursion into the work of Morawski and Smyth, we can easily recognize the constrained perspective the received view provides. It almost feels like it stops precisely where the fruitful analyses of the psychologist‐historians begin.

## Attempt at an Integration as a Conclusion

This article examined the received view of psychological textbooks in the journal *Teaching of Psychology*. I argued that this standard view is quite different than the trends in wider history of science when talking about science textbooks. The difference is not a consequence of the research object—psychological textbook versus other science textbooks—but rather a consequence of psychologists describing textbooks for an audience of psychologists, not historians. The psychologists doing this work were the authors publishing about introductory (and other) textbooks in *Teaching of Psychology*. What made the approach of these teachers of psychology into a history was Weiten and Wight's chapter in *Teaching of Psychology in America: A History*. The chapter recast the products of the teachers of psychology writing about textbooks into a history, transferring it from the context of pedagogical investigations of textbooks to a full‐blown history of these textbooks. What was aimed to tell us more about textbook selection procedures by instructors, content representations and misrepresentations, and other points of interests of the teachers; suddenly became a story describing the change and role of textbooks through time. A history of this sort is a history told for the audience of those teachers in the first place, for whose research it serves as an overview.

The received view falls flat as a history in the professional sense when compared to the work of Morawski and Smyth precisely because these authors write for historians (or psychologist‐historians). The rich descriptions of contexts, be it social or intellectual or both, the massaging of the minutiae of particular discourses, the tracing of the construction of objects/subjects or viewpoints, the disciplinary negotiations, and descriptions of boundary‐work are all blatantly missing from the received view.

More specifically, what is the difference between these two conceptions of textbooks, the alternative and the received? One is a site of fact making versus an aggregator of facts; a place for constructing subjectivities versus just describing them; a context of a discipline versus a portrait of a discipline; a product engaged performatively with its context versus a text consisting of numeric‐variabilized characteristics; a boundary‐object versus a manual of the discipline. It is relatively easy to argue that they are radically different and those differences are insurmountable—go about it as Leahy (2002) did in his scathing criticism of (a few particular) psychologists writing history. In an oversimplification: psychologists write bad histories of their discipline, quantifying left and right, and we need historians to explain what is what in a nuanced and sensitive way.

I do not think the above is the case. Among other things, this article serves as an argument precisely against such scholastic distinctions—the citational analysis used to explore the work published in *Teaching of Psychology* is a case in point. I endorse the view that both the received view and the alternative need to be put side by side, or better said, step after step, in defining textbooks as historiographical objects.

By doing that, textbooks become historically constituted sites of fact making and constructing subjectivities, acting as boundary‐objects between laymen and experts. Moreover, these historiographical objects are represented and described by actors functioning within the context, or a family‐related one, those textbooks describe in the first place. The received view shows us that: How the discipline of psychology in the 1970s a historian would try to understand, for example, suddenly becomes the very part of textbook historiography through the work of Quereshi, mediated by Weiten and Wight. The psychologists’ practices got extended into historical writing about psychology textbooks, collapsing the border between historiography and history. If we recognize the received view as a history of psychology, then our object is not just the textbook, but the textbook plus its surrounding historiography.

By conceptualizing it like that, we make evident the interplay between psychologists writing textbooks about the discipline that at the same time frames their way of thinking. One has to extend the circle to include psychologists writing about psychologists writing textbooks. A perspective like this integrates the received view of Weiten and Wight with the different readings aimed at other audiences than just psychologists. We can recognize their quantification of textbooks as one of the legitimate practices of the historian, but we cannot see it as the same thing historians do when they quantify—the quantification in Weiten and Wight's chapter is the consequence of a research practice being transplanted from the literature on teaching of psychology. There is no evaluative judgement here—I think there is no point in following Leahy in saying A is bad history, while B is good history (instead of good one could also say correct/truthful/objective and still miss the point). Both are histories aimed at different audiences, written with different goals in mind, and we should use both to come to a historiography of textbooks.

Integrating historiographies is not such a radical suggestion, especially if we consider it is not without precedent. On the contrary, in the larger context of history of psychology, the methodological bifurcation in textbook historiography between psychologists and psychologist‐historians is the odd one out. The psychologists’ toolkit—arithmetization in Danziger's words, the language of variables and statistics—already has a place as one of the ways of creating historical arguments. Danziger and Dzinas's (1997) investigation of psychology's metalanguage of variables is an example of such a content analysis (applying it to journals, not textbooks) used to make a historical claim. We can see similar methods used in Andrew Winston's (2004) investigation of the same metalanguage in textbooks. Extending it even further, large scale analyses of texts are a veritable research field in history of psychology, with pioneers such as Christopher Green (e.g., Green & Feinerer, 2015; Green, Feinerer, & Burman, 2015). Obviously, the point I am trying to make is not an all‐out rejection of quantitative analysis in history of psychology, or in the history of introductory textbooks in psychology.

It feels like the received view made the first step of generating content, but then never interpreted it historically. The textbooks just remained the very minimalistic description in the terms of the already established metalanguage of psychology. The force of the numbers was used to buttress a simple chronology, and not a contextualized and reflexive account of the role and function of textbooks in psychology.

One possible venue of integration is recasting my account of textbook historiographies into Latour's (2005) actor‐network theory (ANT)—looking at textbooks as pieces in an expanding network of associations. Textbooks get transferred and transplanted from one context into another, reformed and recast with various meanings. Describing it in these terms, we could look at the integrated historiography being the result of three translations—the first when the textbook was taken from the classroom and the market by the authors publishing in *Teaching of Psychology* from the 1970s onward, who tried to optimize its selection and content with statistical methods. Then this expanding body of research consisting of hundreds of articles in our citation analysis were translated and synthesized into a history of those textbooks—the textbooks have turned from an optimizable and faulty receptacle of facts into a series of portraits of a discipline, effectively glimpses into the essential discipline of psychology. The last transfer was conducted in this article, where these portraits of a discipline crafted by Weiten and Weight were downplayed by describing their construction of subjectivities (through their content) and objectivity (through their fact‐making techniques) endorsed by psychologist‐historians.

Every translation has changed the textbooks—the first refashioning them into objects susceptible to statistics, the second using these quantitatively represented objects as windows into history, and the third designating these windows as a stepping stone for historical interpretation. Keeping with the Latourian allegory, the thousands of psychology textbooks sketchily marched through assemblages of psychologists and their statistical methods, psychologist‐historians, historians, journals (and their audiences), editors, and edited volumes. The textbook that existed before authors in *Teaching of Psychology* started writing about them, and the ones we have at the end of this article are almost nothing alike.

I would keep this ANT interpretation as an allegory that helps us understand the argument in the article, but not a definite conclusion. After all, particular textbooks were hardly dealt with in the article, and the intricacies of ANT's descriptions of actors and their movements through the network would needlessly overburden the whole argument. It also chronologically misrepresents the actual research on textbooks—what is called the received view still thrives in *Teaching of Psychology* as part of research on the classrooms where psychology is taught; the articles and the textbooks constructed in them are quite unscathed by what was called Weiten and Wight's translation in our allegory.

The thesis I sketched in the introduction—that the received view of textbooks in psychology is different from the view of textbooks in wider history of science still stands firm. The textbook as an object produced by the publications in *Teaching of Psychology* is something radically different than the object Morawski, Smyth, and Olesko describe in their work. However, my account of the received view was not produced to criticize this difference—agreeing with, for example, Morawski's view of the textbook at the expense of the one produced by Weiten. It was aimed to produce a textbook as a historiographical object that bridges this chasm between the standard view and its counterpart.

Casting it Hacking's terminology (2002), the historiography of psychological textbooks should follow their historical ontologies by traveling through various contexts—the ones they were written in (author's), the ones they were written for (classrooms), and the ones they were debated in (among psychologists and historians). The textbook, as a nexus of all kinds of forces—of the academic community (both of psychologists and historians), of the pedagogical necessity, marketing of the discipline's practical utility, authors idiosyncrasies, the interest of the publishers—is too fruitful to be left to just content analysis, or just discourse analysis.

Trying to find one last lesson from this search through the muddled and disconnected scholarship of textbooks, I would argue that this makes psychological textbooks enter center stage of historiography of science through the backdoor. From a marginal historical space confined to science pedagogy and book history, it offers itself instead as one of the possible platforms for debates of disciplinary formation, fragmentation, and identity. Textbooks become reifications of investigative practices coupled with pedagogical ones. They mesh the contexts of theoretical knowledge and application, the rhetoric of presenting it, the business of teaching it, and provide objective and objectified perspectives on a discipline, perspectives that can be carefully recast and inspected. They are full of “facts” that at the same time bring and hide uncertainties—they are textual witnesses of a time gone by. Textbooks (again?) become a place to go to reflect about the discipline. A good Latourian might even say textbooks gain agency in driving that reflection.

## Footnotes

1 The textbook's goal of constructing a subjectivity that is at odds with commonsense is precisely what Morawski (1996) aims at: “Psychology's success in undermining commonsense knowledge and in marketing an apparently unsavory model of subjectivity depended on the reader's dissociation from that subjectivity” (p. 153). This notion of waging war on the commonsense understanding of the world signals the difficult position psychology is in as a scientific discipline. If we compare it to W. V. O. Quine's (1951, p. 42) notion of “science [as] an extension of common sense,” no wonder that “patrolling the borders of psychology” (Smyth, 2001b) as a scientific discipline takes so much effort and specific strategies. It serves as epistemological legitimization; in other words, psychology is either scientific/counterintuitive or commonsensical/trivial.

2 The line could be drawn by demarcating who produced the histories, but making the distinction between the aimed audiences of the histories is much more fruitful in this case, because both Jill Morawski and Mary Smyth are psychologists by training, but they argue quite different points than the psychologists publishing in *Teaching of Psychology*. The difference stems precisely out of the different questions they ask. These questions are provided with answers that would interest a specific audience, in this case, of historians of science. The audience makes all the difference for the type of argument one tries to construct and make believable, as will be argued in this article. So instead of saying “psychologists’ history of textbooks” and “historians’ history of textbooks,” it would be better to say the history of textbooks for psychologists, and the history of textbooks for historians, respectively.

3 For a nonessentialist historical approach, see Thomas Teo's (2007) treatment of German introductory psychology texts in nineteenth century Germany.

4 The argument that we sometimes constitute an object of research with the way we research it bears resemblance to Gerd Gigerenzer's (1991) tools‐to‐theory heuristic. The case Gigerenzer makes is that some cognitive models of the human mind are based on inferential methods. In our case, the example is much less lofty—the descriptive statistics of topical coverage and factor analytically derived categorizations are what the textbook becomes, at least as a historical object.
